# Assessment of temperatures in the vaccine cold chain in two provinces in Lao People’s Democratic Republic: a cross-sectional pilot study

**DOI:** 10.1186/s13104-018-3362-1

**Published:** 2018-04-27

**Authors:** Tomomi Kitamura, Viraneth Bouakhasith, Kongxay Phounphenghack, Chansay Pathammavong, Anonh Xeuatvongsa, Masataro Norizuki, Hironori Okabayashi, Yoshio Mori, Munehito Machida, Masahiko Hachiya

**Affiliations:** 1 Bureau of International Cooperation, National Center for Global Health and Medicine, 1-21-1 Toyama, Shinjuku, Tokyo 1628655 Japan; 2 National Immunization Program, Ministry of Health, Lao PDR, Simuang Road, Vientiane, Lao People’s Democratic Republic; 3 Laboratory of Rubella, Department of Virology III, National Institute of Infectious Diseases, Murayama Branch, 4-7-1 Gakuen, Musashimurayama, Tokyo 208-0011 Japan; 4 Department of Global Health, Faculty of Medicine, Institute of Medical, Pharmaceutical and Health Sciences, Kanazawa University, 13-1 Takaramachi, Kanazawa, Ishikawa 9208640 Japan

**Keywords:** Vaccines, Cold chain, Storage, Transportation, Temperature

## Abstract

**Objective:**

All childhood vaccines, except the oral polio vaccine, should be kept at 2–8 °C, since the vaccine potency can be damaged by heat or freezing temperature. A temperature monitoring study conducted in 2008–2009 reported challenges in cold chain management from the provincial level downwards. The present cross-sectional pilot study aimed to assess the current status of the cold chain in two provinces (Saravan and Xayabouly) of Lao People’s Democratic Republic between March–April 2016. Two types of temperature data loggers recorded the temperatures and the proportions of time exposed to < 0 or > 8 °C were calculated.

**Results:**

The temperature remained within the appropriate range in the central and provincial storages. However, the vaccines were frequently exposed to > 8 °C in Saravan and < 0 °C in Xayabouly in the district storage. Vaccines were exposed to > 8 °C during the transportation in Saravan and to both > 8 and < 0 °C in Xayabouly. Thus, challenges in managing the cold chain in the district storage and during transportation remain, despite improvements at the provincial storage. A detailed up-to-date nationwide analysis of the current situation of the cold chain is warranted to identify the most appropriate intervention to tackle the remaining challenges.

## Introduction

Immunization is one of the most important achievements in public health, and a major contributor for this success is the expanded program on immunization (EPI) [1–14]. The EPI was initiated in Lao People’s Democratic Republic (Lao PDR) in 1979, and the immunization coverage has been improving since then; however, the country has experienced outbreaks of vaccine-preventable diseases lately, and one of the reasons was speculated as several steps of the cold chain potentially being compromised [15–18].

The World Health Organization and vaccine labels state that all childhood vaccines, except for oral polio vaccines, should be kept at 2–8 °C to ensure their quality, efficacy, and safety, since most vaccines are sensitive to heat or freezing temperatures [10–13, 19]. In Lao PDR, the supply chain consists of three levels. The vaccines are dispatched from the National Immunization Program (NIP) storage in the capital to the regional or provincial storage. Subsequently, they are sent to the district storage. Finally, they reach the health centers. Immunization services are provided either at the health facilities or during outreach sessions in the villages. A temperature monitoring study conducted in 2008–2009 by the United Nations Children’s Fund (UNICEF) reported several challenges in cold chain management from the provincial level downward and the vaccines were exposed both overheating and freezing [20]. A new strategy has been implemented such as continuous monitoring at storage since then. With this in mind, the present study and aimed to assess the current status of the cold chain, at all levels, in two representative provinces of Lao PDR.

## Methods

**Fig. 1:**
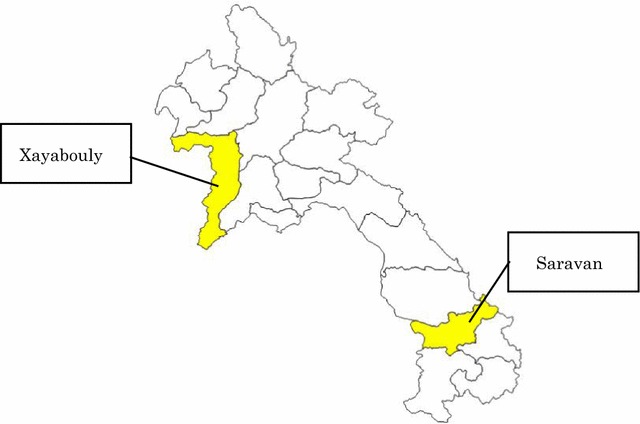
Map of Lao PDR showing the target two provinces https://gadm.org/maps/LAO_1.html

This was a cross-sectional pilot study conducted from March to April 2016. Two provinces, one each in the northern and southern part of Lao PDR, were deliberately selected by the NIP since these two provinces are good representatives of all 18 provinces considering their population and geographical characteristics (Fig. 1). One district per province and five health centers per district were selected by the EPI officers of the NIP and the Provincial Health Department from each province. Sampling method is a purposive sampling and sample size was justified since this study tested feasibility and acceptability of the study protocol for a larger study in the future. The ambient temperature was not recorded in this study to avoid the complexity of the procedure for the health care workers. DTP-HepB-Hib vaccines was agreed to transport through a normal vaccine transportation route among the researchers before the study was conducted.

Two types of temperature data loggers were used and randomly assigned to the final destination: MicroLite™ (Sato Shouji Inc., Japan) and CUSTOM™ (CUSTOM, Japan). MicroLite™ was pre-set to record temperatures every two minutes and CUSTOM™ was set to record every 5 min. The health care workers were all pre-informed about the study, however they were not informed about the monitoring intervals and the loggers were pre-set to avoid manipulations by the health care workers to minimize the bias. The recordings were started on 17/3/2016 and lasted until 11/4/2016. All data loggers and vaccines started to travel down to the provinces on 24/3/2016. A monitoring form was developed and attached with the vaccines and temperature data loggers to record the dates of events such as the arrival and departure of the vaccines. The provincial/district EPI officers and the health center staff recorded the information in the forms. The temperature data loggers and monitoring forms were packed with the vaccines in the vaccine carriers and transported using the normal transportation route. Once they reached the provincial/district storage, they were stored in the refrigerator with the vaccines until they were ready to be dispatched to the lower level.

All data were downloaded from each temperature data logger as an Excel spreadsheet or PDF file. The analysis conducted in the study was descriptive and the proportions of time exposed to < 0 and > 8 °C were calculated. The precise times (hours or minutes) of arrival and departure were not recorded, and the duration of transportation was defined from 0:00 on the departure date to 23:59 on the arrival date. The duration of storage was defined as 24 h per date not indicated as being in transportation.

### Ethical considerations

No ethical clearance or informed consent was necessary, since there was no intervention or interaction with humans [10]. However, authorization to conduct this study was granted by the Ministry of Health and the Provincial Health Departments [10].

## Results

Eight and six days of recordings were analyzed for the loggers that went to Saravan (23/3/2016–31/3/2016) and Xayabouly (23/3/2016–29/3/2016), respectively.

### Ambient temperature and range of temperature during the study period

Ambient temperature was not recorded in this study. The temperature ranged from 19 to 38 °C in Vientiane, capital of Lao PDR, during the study period. The temperature in Pakse and Champasak, which are located 54 km from Saravan, ranged from 21 to 37 °C, while the recorded temperatures in Saravan ranged between 0.2 and 18.0 °C; these were below the ambient temperature recorded in Pakse. The temperature in Luang Prabang, which is located 49 km from Xayabouly, ranged from 18 to 35 °C. In Xayabouly, the temperature ranged between − 3.3 and 20.4 °C [21].

### Proportions of exposure to suboptimal temperature throughout the observation period

On average, the vaccines were exposed to > 8 °C for 41% of the total recorded time in Saravan (37–45%). They were not exposed to < 0 °C. The corresponding proportions in Xayabouly were 5% (5–6%) and 15% (11–19%), respectively.

### Proportions of exposure to suboptimal temperature during storage

No exposure to suboptimal temperature in the central storage was observed 1 day prior to the vaccines and data loggers being dispatched (Table 1). The times spent at the provincial and district storages were 2 days in Saravan and 1 day in Xayabouly. No exposure to suboptimal temperature was seen in the provincial storage in both provinces. In Saravan, the loggers recorded more than 80% of the time spent in the storage as being at above 8 °C (range of proportion: 84–100%, range of temperature: 7.9–11.9 °C), whereas there was no exposure to < 0 °C at the district storage. In Xayabouly, no exposure to > 8 °C was recorded; however, more than 50% of the time, the temperature was recorded as < 0 °C (range of proportion: 44–68%, range of temperature: − 2.7 to 6.3 °C) at the district storage.

**Table 1:** Suboptimal temperatures in the cold-chain system during storage

Province, district, health centre (data logger)	Date of storage					
	Central		Province		District	
	Proportion of time spent at temperature (%)					
	> 8 °C	< 0 °C	> 8 °C	< 0 °C	> 8 °C	< 0 °C
Saravan province	23/3/2017		26/3/2017–27/3/2016		29/3/2016–30/3/2016	
Laogham, Onnoi (MicroLite™), %	0	0	0	0	100	0
	Max 6.7 °C, min 5.3 °C		Max 6.0 °C, min 5.8 °C		Max 11.9 °C, min 8.1 °C	
Laogham, Dasia (MicroLite™), %	0	0	0	0	100	0
	Max 6.7 °C, min 5.0 °C		Max 5.8 °C, min 5.4 °C		Max 10.4 °C, min 8.1 °C	
Laogham, Nongkae (MicroLite™), %	0	0	0	0	100	0
	Max 6.7 °C, min 5.2 °C		Max 6.0 °C, min 5.7 °C		Max 10.8 °C, min 8.3 °C	
Laogham, Dongyai (CUSTOM™), %	0	0	0	0	85	0
	Max 6.5 °C, min 4.6 °C		Max 5.8 °C, min 5.5 °C		Max 11.5 °C, min 7.9 °C	
Laogham, Naborn (CUSTOM™), %	0	0	0	0	84	0
	Max 6.4 °C, min 4.8 °C		Max 5.7 °C, min 5.4 °C		Max 10.5 °C, min 7.9 °C	
Xayabouly province	23/3/2017		26/3/2017		28/3/2016	
Kanethao, Huayrot (MicroLite™), %	0	0	0	0	0	44
	Max 6.8 °C, min 5.3 °C		Max 4.4 °C, min 3.9 °C		Max 6.1 °C, min − 1.5 °C	
Kanethao, Vungpa (MicroLite™), %	0	0	0	0	0	51
	Max 6.6 °C, min 5.1 °C		Max 4.3 °C, min 3.8 °C		Max 5.9 °C, min − 2.7 °C	
Kanethao, Nahin (CUSTOM™), %	0	0	0	0	0	68
	Max 6.7 °C, min 4.9 °C		Max 4.1 °C, min 3.6 °C		Max 6.0 °C, min − 2 °C	
Kanethao, Pakkham (CUSTOM™), %	0	0	0	0	0	68
	Max 6.4 °C, min 4.8 °C		Max 4.1 °C, min 3.5 °C		Max 4.9 °C, min − 2.4 °C	
Kanethao, Huayleru (CUSTOM™), %	0	0	0	0	0	54
	Max 6.5 °C, min 4.7 °C		Max 4.1 °C, min 3.6 °C		Max 6.3 °C, min − 2.0 °C	

### Proportions of exposure to suboptimal temperature during transportation

The times required to transport the vaccines from the capital to the province, from the province to the district, and from the district to the health centers were 2, 1 and 1 day, respectively, in both provinces (Table 2). The vaccines were exposed to > 8 °C at an average of 48% (45–50%), 51% (41–61%), and 32% (27–39%) of the time spent for transportation from the capital to province, from province to district, and from district to health center, respectively in Saravan. They were never exposed to < 0 °C. The differences in maximum and minimum temperature were 11.0, 11.4 and 17.8 °C respectively (capital → province: maximum 15.5 °C, minimum 4.5 °C; province → district: max 16.8 °C, min 5.4 °C; district → health center: max 18.0 °C, min 0.2 °C). The vaccines were exposed to > 8 °C at an average of 6% (5–10%), 18% (13–25%), and 8% (2–11%) of the time during transportation in Xayabouly and to < 0 °C at an average of 0%, 12% (9–17%), and 32% (8–51%) of the time during transportation from the capital to province, from province to district, and from district to health center, respectively. The ranges of temperature were 16.8, 15.8 and 23.7 °C, respectively (capital → province: max 18.4 °C, min 1.6 °C; province → district: max 14.3 °C, min –2.7 °C; district → health center: max 20.4 °C, min − 3.3 °C).

**Table 2:** Suboptimal temperatures in the cold-chain system during transportation

Province, district, health centre (data logger)	Date of transportation					
	Capital → province		Province → district		District → health centre	
	Proportion of time spent at temperature (%)					
	> 8 °C	< 0 °C	> 8 °C	< 0 °C	> 8 °C	< 0 °C
Saravan province	24/3/17–25/3/17		28/3/2017		31/3/2017	
Laogham, Onnoi (MicroLite™), %	49	0	60	0	29	0
	Max 13.9 °C, min 5.1 °C		Max 16.6 °C, min 5.8 °C		Max 9.4 °C, min 2.1 °C	
Laogham, Dasia (MicroLite™), %	50	0	45	0	27	0
	Max 15.0 °C, min 4.9 °C		Max 16.3 °C, min 5.4 °C		Max 8.9 °C, min 0.9 °C	
Laogham, Nongkae (MicroLite™), %	50	0	61		39	0
	Max 15.4 °C, min 5.0 °C		Max 16.3 °C, min 5.7 °C		Max 9.8 °C, min 0.2 °C	
Laogham, Dongyai (CUSTOM™), %	45	0	41	0	29	0
	Max 12.9 °C, min 4.5 °C		Max 15.9 °C, min 5.6 °C		Max 10.8 °C, min 0.9 °C	
Laogham, Naborn (CUSTOM™), %	47	0	45	0	34	0
	Max 15.5 °C, min 4.6 °C		Max 16.8 °C, min 5.5 °C		Max 18.0 °C, min 0.9 °C	
Xayabouly province	24/3/17–25/3/17		27/3/2017		29/3/2017	
Kanethao, Huayrot (MicroLite™), %	10	0	13	9	7	25
	Max 15.4 °C, min 3.0 °C		Max 11.6 °C, min − 1.3 °C		Max 10.1 °C, min − 1.0 °C	
Kanethao, Vungpa (MicroLite™), %	7	0	13	17	10	8
	Max 15.2 °C, min 3.0 °C		Max 11.3 °C, min − 2.7 °C		Max 11.8 °C, min − 0.2 °C	
Kanethao, Nahin (CUSTOM™), %	6	0	24	11	2	51
	Max 16.5 °C, min 1.6 °C		Max 13.5 °C, min − 1.7 °C		Max 10.5 °C, min − 3.3 °C	
Kanethao, Pakkham (CUSTOM™), %	5	0	15	13	11	49
	Max 14.4 °C, min 2.3 °C		Max 13.3 °C, min − 1.9 °C		Max 10.2 °C, min − 3.1 °C	
Kanethao, Huayleru (CUSTOM™), %	5	0	25	11	9	28
	Max 18.4 °C, min 2.6 °C		Max 14.3 °C, min − 1.7 °C		Max 20.4 °C, min − 1.3 °C	

## Discussion

This pilot study aimed to identify the current temperature control of vaccines in Lao PDR. This study discovered challenges during the storage at the district and during transportation, and these challenges differed according to the region. The vaccines were exposed to both overheating and freezing.

The study of temperature monitoring in 2008–2009 by UNICEF pointed out significant weaknesses of cold chains in terms of material and human resources from the provincial level downwards, including improperly maintained or outdated refrigeration equipment, poor compliance with cold-chain procedures, inadequate monitoring, and poor understanding of the dangers of freezing vaccines [20]. The present study showed that the temperature remained within the appropriate range in the provincial level storage. However, at the district storage, the vaccines were still exposed to either overheating or freezing during a high proportion of the time spent there. The temperature exceeded 5 °C above or below the appropriate temperature range. Besides the weaknesses pointed out by UNICEF, the studies from five different countries described an unstable power supply or lack of contingency plans during power cuts a lack of training on the cold chain, insufficient supervision to implement proper monitoring, lack of access to guidelines or Standard Operating Procedures, and improper management of the budget for the cold chain might be root causes of inappropriate cold chain management below the district level [10, 11, 13, 14, 19, 20, 22].

In the present study, the vaccines were exposed to either overheating or freezing throughout the transportation from the central to the health centres as well, however the differences in the maximum and minimum temperature was greater than 10 °C, suggesting that the temperature control during transport was more unstable than that in storage. In addition to the reasons mentioned above, the studies from four different countries described the characteristic reasons during transport as unstable temperature control during transportation might be due to improper management of ice packs, unexpected delays in transportation due to road or vehicle conditions, and improper means of transporting vaccine [10, 11, 13, 19, 22]. The study by Nanthavong et al. mentioned the possibility of cold chain breaks during transport in Lao PDR [17].

Emphasis has long been placed on avoiding high temperatures, but, as this study also showed, recent studies have reported exposure to freezing temperature at many stages during distribution [12, 23, 24]. The World Health Organization guidelines specify that the hepatitis B, diphtheria–tetanus–pertussis, diphtheria–tetanus, and tetanus toxoid vaccine must not be frozen [12]. To avoid freezing of vaccines, Kolwaite et al. conducted a pilot study to assess the effect of “out-of-the-cold-chain” storage in Lao PDR and discovered improved Hepatitis B dose coverage without an increase in adverse reactions [25]. Some innovative solutions should be considered in the context of developing countries, such as a computerizing temperature monitoring system, development of thermostable vaccines, development of a compact prefilled vaccination device, or the use of a cold box that can manage temperature control on its own for a long duration [10, 14, 24, 26].

## Conclusion

This study identified the current status of cold chain management for vaccines in two provinces in Lao PDR. Despite improvements in the cold chain management at the provincial storage, it remains a challenge to manage the cold chain in the district storage and during transportation. Both overheating and freezing of vaccines were identified, and these findings differed according to the region. A detailed up-to-date analysis of the current situation of the cold chain at all levels is warranted in Lao PDR, such as a nationwide cold chain assessment to implement proper context-specific interventions for different cold chain management issues.

## Limitations

This study has several limitations. First, this study only covered two out of 18 provinces. Second, the ambient temperature was not recorded; however, we speculate that the temperature loggers were travelled or stored in the vaccine carriers or refrigerator as instructed, since the available temperatures from the neighboring provinces were higher than those recorded in the loggers. Third, the temperature management in the health center storages and during the outreach activities were not monitored. Fourth, the precise times of arrival and departure were not recorded and the transportation period may thus also include time spent in the storage. Finally, all health care workers were conscious that the temperature was being monitored, thus, their vaccine handling may have been changed. The results should be carefully interpreted by considering these limitations.

## Abbreviations

EPI: expanded program on immunization
Lao PDR: Lao People’s Democratic Republic
NIP: National Immunization Program
UNICEF: United Nations Children’s Fund

## References

[1] Muhsen K, Abed El-Hai R, Amit-Aharon A, Nehama H, Gondia M, Davidovitch N (2012). Risk factors of underutilization of childhood immunizations in ultraorthodox Jewish communities in Israel despite high access to health care services. Vaccine..

[2] Rodewald L, Maes E, Stevenson J, Lyons B, Stokley S, Szilagyi P (1999). Immunization performance measurement in a changing immunization environment. Pediatrics.

[3] Koumare AK, Traore D, Haidara F, Sissoko F, Traore I, Drame S (2009). Evaluation of immunization coverage within the expanded program on immunization in Kita Circle, Mali: a cross-sectional survey. BMC Int Health Hum Rights..

[4] Linkins RW, Salmon DA, Omer SB, Pan WK, Stokley S, Halsey NA (2006). Support for immunization registries among parents of vaccinated and unvaccinated school-aged children: a case control study. BMC Public Health..

[5] Odusanya OO, Alufohai EF, Meurice FP, Ahonkhai VI (2008). Determinants of vaccination coverage in rural Nigeria. BMC Public Health..

[6] Jamil K, Bhuiya A, Streatfield K, Chakrabarty N (1999). The immunization programme in Bangladesh: impressive gains in coverage, but gaps remain. Health Policy Plan..

[7] Xie J, Dow WH (2005). Longitudinal study of child immunization determinants in China. Soc Sci Med.

[8] Kitamura T, Komada K, Xeuatvongsa A, Hachiya M (2013). Factors affecting childhood immunization in Lao People’s Democratic Republic: a cross-sectional study from nationwide, population-based, multistage cluster sampling. Biosci Trends..

[9] Rainey JJ, Watkins M, Ryman TK, Sandhu P, Bo A, Banerjee K (2011). Reasons related to non-vaccination and under-vaccination of children in low and middle income countries: findings from a systematic review of the published literature, 1999–2009. Vaccine..

[10] Ateudjieu J, Kenfack B, Nkontchou BW, Demanou M (2013). Program on immunization and cold chain monitoring: the status in eight health districts in Cameroon. BMC Res Notes..

[11] Yakum MN, Ateudjieu J, Walter EA, Watcho P (2015). Vaccine storage and cold chain monitoring in the North West region of Cameroon: a cross sectional study. BMC Res Notes..

[12] Nelson CM, Wibisono H, Purwanto H, Mansyur I, Moniaga V, Widjaya A (2004). Hepatitis B vaccine freezing in the Indonesian cold chain: evidence and solutions. Bull World Health Organ.

[13] Murhekar MV, Dutta S, Kapoor AN, Bitragunta S, Dodum R, Ghosh P (2013). Frequent exposure to suboptimal temperatures in vaccine cold-chain system in India: results of temperature monitoring in 10 states. Bull World Health Organ.

[14] Anderson R, Perrier T, Pervaiz F, Sisouveth N, Kumar B, Phongphila S, et al., editors. Supporting immunization programs with improved vaccine cold chain information systems. In: IEEE global humanitarian technology conference (GHTC 2014); 2014. 10–13 Oct. 2014.

[15] World Health Organization (2012). International review of the expanded programme on immunization in the Lao People’s Democratic Republic.

[16] World Health Organization. Lao People’s Democratic Republic: WHO and UNICEF estimates of immunization coverage: 2016 revision. 2016. http://www.who.int/immunization/monitoring_surveillance/data/lao.pdf. Accessed 31 Aug 2017.

[17] Nanthavong N, Black AP, Nouanthong P, Souvannaso C, Vilivong K, Muller CP (2015). Diphtheria in Lao PDR: insufficient coverage or ineffective vaccine?. PLoS ONE.

[18] WHO. Circulating vaccine-derived poliovirus—Lao People’s Democratic Republic 2016. 2016. http://www.who.int/csr/don/25-february-2016-polio-lao/en/. Accessed 31 Aug 2017.

[19] Berhane Y, Demissie M (2000). Cold chain status at immunisation centres in Ethiopia. East Afr Med J.

[20] Garnet A (2010). Report on the 2008–2009 temperature monitoring study in Lao PDR.

[21] Time and Date AS. World temperatures—weather around the world. 2017. https://www.timeanddate.com/weather/. Accessed 31 Aug 2017.

[22] Hanjeet K, Lye MS, Sinniah M, Schnur A (1996). Evaluation of cold chain monitoring in Kelantan, Malaysia. Bull World Health Organ..

[23] Lydon P, Raubenheimer T, Arnot-Krüger M, Zaffran M (2015). Outsourcing vaccine logistics to the private sector: the evidence and lessons learned from the Western Cape Province in South-Africa. Vaccine..

[24] Kristensen DD, Lorenson T, Bartholomew K, Villadiego S (2016). Can thermostable vaccines help address cold-chain challenges? Results from stakeholder interviews in six low- and middle-income Countries. Vaccine..

[25] Kolwaite AR, Xeuatvongsa A, Ramirez-Gonzalez A, Wannemuehler K, Vongxay V, Vilayvone V (2016). Hepatitis B vaccine stored outside the cold chain setting: a pilot study in rural Lao PDR. Vaccine..

[26] Guillermet E, Dicko HM, le Mai TP, N’Diaye M, Hane F, Ba SO (2015). Acceptability and feasibility of delivering pentavalent vaccines in a compact, prefilled, autodisable device in Vietnam and Senegal. PLoS ONE.

